# Medicinal cannabis for psychiatric disorders: a clinically-focused systematic review

**DOI:** 10.1186/s12888-019-2409-8

**Published:** 2020-01-16

**Authors:** Jerome Sarris, Justin Sinclair, Diana Karamacoska, Maggie Davidson, Joseph Firth

**Affiliations:** 1 NICM Health Research Institute, Western Sydney University, Westmead, NSW 2145 Australia; 2 Department of Psychiatry, The Melbourne Clinic, Professorial Unit, The University of Melbourne, Melbourne, Australia; 3 Division of Psychology and Mental Health, University of Manchester, Manchester, UK

**Keywords:** Cannabinoids, Cannabidiol, Marijuana, Cannabis, CBD, THC, Pharmacogenomics, Medicinal plants, Mental health

## Abstract

**Background:**

Medicinal cannabis has received increased research attention over recent years due to loosening global regulatory changes. Medicinal cannabis has been reported to have potential efficacy in reducing pain, muscle spasticity, chemotherapy-induced nausea and vomiting, and intractable childhood epilepsy. Yet its potential application in the field of psychiatry is lesser known.

**Methods:**

The first clinically-focused systematic review on the emerging medical application of cannabis across all major psychiatric disorders was conducted. Current evidence regarding whole plant formulations and plant-derived cannabinoid isolates in mood, anxiety, sleep, psychotic disorders and attention deficit/hyperactivity disorder (ADHD) is discussed; while also detailing clinical prescription considerations (including pharmacogenomics), occupational and public health elements, and future research recommendations. The systematic review of the literature was conducted during 2019, assessing the data from all case studies and clinical trials involving medicinal cannabis or plant-derived isolates for all major psychiatric disorders (neurological conditions and pain were omitted).

**Results:**

The present evidence in the emerging field of cannabinoid therapeutics in psychiatry is nascent, and thereby it is currently premature to recommend cannabinoid-based interventions. Isolated positive studies have, however, revealed tentative support for cannabinoids (namely cannabidiol; CBD) for reducing social anxiety; with mixed (mainly positive) evidence for adjunctive use in schizophrenia. Case studies suggest that medicinal cannabis may be beneficial for improving sleep and post-traumatic stress disorder, however evidence is currently weak. Preliminary research findings indicate no benefit for depression from high delta-9 tetrahydrocannabinol (THC) therapeutics, or for CBD in mania. One isolated study indicates some potential efficacy for an oral cannabinoid/terpene combination in ADHD. Clinical prescriptive consideration involves caution in the use of high-THC formulations (avoidance in youth, and in people with anxiety or psychotic disorders), gradual titration, regular assessment, and caution in cardiovascular and respiratory disorders, pregnancy and breast-feeding.

**Conclusions:**

There is currently encouraging, albeit embryonic, evidence for medicinal cannabis in the treatment of a range of psychiatric disorders. Supportive findings are emerging for some key isolates, however, clinicians need to be mindful of a range of prescriptive and occupational safety considerations, especially if initiating higher dose THC formulas.

## Introduction

The Cannabaceae family is a comparatively small family of flowering plants encompassing 11 genera and approximately 170 different species, a small number of which elicit a range of varying psychoactive effects [1]. Several medical applications have been studied over the past decades, with the National Academies of Sciences, Engineering and Medicine (NASEM) recently holding the position that cannabis and cannabinoids demonstrate conclusive or substantial evidence for chronic pain in adults, chemotherapy-induced nausea and vomiting and spasticity in multiple sclerosis, with limited evidence for use in increasing appetite in HIV/AIDS patients and improving symptoms of post-traumatic stress disorder (PTSD) [2].

While there is increasing psychiatric interest (and debate) regarding the potential mental health applications (in concert with concerns over the potential for triggering latent psychosis), historical evidence for the use of cannabis in mental health conditions is remarkably ancient. For instance, the *Shen-nung Pen-tsao Ching* (Divine Husbandman’s *Materia Medica*) described its benefit as an anti-senility agent [3, 4], while in the Assyrian culture, cannabis was indicated as a drug for grief and sorrow [5, 6]. Sections of the Indian *Atharva Veda* (1500 BCE) suggest *bhanga* (Cannabis) exerted anxiolytic effects [5, 7], while in 1563, Da Orta [8] described cannabis as allaying anxiety and engendering laughter. With respect to modern use, contemporary consumers of cannabis report (as assessed via meta-analysis of patient usage data) that pain (64%), anxiety (50%), and depression/mood (34%) are the most common reasons [9].

Increasing scientific research, conducted over recent years, has seen the regulatory pendulum swinging away from the United Nations Single Convention on Narcotic Drugs in 1961 (which recommends enforcement of cannabis use as illegal) [10], towards consideration of its potential use in medical conditions. Recent scientific evidence ascribes anxiolytic, neuroprotective, antioxidant, anti-inflammatory, antidepressant, anti-psychotic and hypnotic pharmacological actions due to several phytochemicals commonly found in the cannabis genus [11, 12].

While Δ^9^-tetrahydrocannabinol (THC) is considered the main psychoactive constituent, other cannabinoids have also revealed less potent psychotropic effects. These include cannabidiol (CBD) [13], Δ^8^-tetrahydrocannabinol [14], and other less-studied cannabinoids including cannabinol (CBN) and Δ^8^ tetrahydrocannabivarin (THCV) [15]. Further, many other constituents such as the terpenes (i.e. volatile organic compounds found mainly as essential oils in many plants), also provoke a range of biological effects, and produce the characteristic aroma of the plant [16]. The hundreds of cannabis chemovars or varieties (commonly referred to as strains) developed over millennia have unique and complex constituent profiles, of which each may provide targeted therapeutic usage due to the unique synergistic combination of plant chemicals. Some pharmaceutical preparations have attempted to isolate the key constituents (there are over 140 phytocannabinoids [17]) to provide standardised formulas that may harness this ‘entourage effect’ [16, 18], while being able to provide batch-to-batch assurance of the medicine.

While other reviews have covered cannabis’ use in a range of conditions (cf. Whiting et al. 2015 [10] for a general review of evidence for medicinal cannabis), none to date have provided both a systematic and ‘clinically-focused’ review on psychiatric disorders. As the focus was on emerging data for the use of mental health disorders, we omitted addiction and neurological disorders, which have been extensively covered elsewhere [10, 19], cf. pain [20–22], cf. epilepsy [23, 24], cf. movement disorders [25]. A further motive for this paper focusing solely on psychiatric disorders, concerns cannabis users noting that self-reported anxiety, insomnia, and depression symptoms are amongst the most common reasons for usage [26].

Thus, the primary purpose of this paper is to provide a systematic review of the current state of evidence in the emerging field of cannabinoid therapies for psychiatric disorders (PTSD, generalised anxiety disorder, social anxiety, insomnia, psychotic disorders, and attention-deficit hyperactivity disorder: ADHD). In addition, this review provides clinical prescriptive guidelines and consideration of both safety and occupational public health issues. We also provide discussion on considerations for future research in the field. Our intention was to provide a review of the extant literature to inform a discussion with clinical context and appropriate recommendations.

## Methods

Due to the field still being in its infancy, a broad inclusion criteria was applied to the available data. The purpose was to locate human studies involving whole cannabis plant medicines and cannabis-derived isolates (singularly or in combinations) for the treatment of major psychiatric disorders or mental health symptoms. Synthetic cannabinoid analogues (e.g. nabilone) and THC isomers (e.g. dronabinol) were omitted as these fall under the auspices of a pharmaceutical-focused review (as these are classified as pharmaceutical ‘drugs’).

Major electronic databases including OVID MEDLINE, Cochrane Central Register of Controlled Trials, Health Technology Assessment Database, Allied and Complementary Medicine and PsychINFO were accessed for data up to July 2019. Initially, data were sought for meta-analytic or systematic review level epidemiological evidence (as there is sufficient data available) on the cross-sectional or longitudinal association of cannabis use and individual psychiatric disorders or symptoms. This was undertaken to assess any deleterious relationship between cannabis and psychiatric disorders. We then specifically sought any literature involving interventional human trials and observational studies, including case studies (due to deficient randomised controlled trials [RCTs] in this emerging area). We included studies with any sample size or age or gender, which used either inhalant, oral, or transdermal administration of medicinal cannabis or cannabis-derived isolates. All studies in English were assessed for inclusion (see supplementary data for the PRISMA flow chart for the number of human clinical trials or case studies excluded/included). The results are presented to firstly cover the major current epidemiological evidence, and then next all available clinical trial or case study data.

The following search terms were used to locate human studies or case report publications:

- TITLE: cannab* OR THC OR tetrahydrocannabinol OR canab*
- AND
- TITLE: depression or depressive or mental illness* or mental disorder* or mental health or mood disorder* or affective disorder* or anxi* or panic disorder or obsessive compulsive or adhd or attention deficit or phobi* or bipolar or psychiat* or psychological or psychosis or psychotic or schizophr* severe mental* or serious mental* or antidepress* or antipsychotic* or post traumatic* or personality disorder* or stress

In summation, 481 articles were located, which was reduced to 310 after duplicates were removed. Of these, 13 studies fitted the eligibility criteria as clinical studies of cannabis-based treatments for symptoms of psychiatric disorders. The full search and screening process is displayed in the supplementary data. There were insufficient homogenous studies to perform a meta-analysis.

### Affective disorders

#### Anxiety

The endocannabinoid system has been found to be a modulator of anxiety and mood, with recent data showing that cannabinoids or substances which target this system may interact with specific brain regions, including the medial prefrontal cortex, amygdaloid complex, bed nucleus of stria terminalis, and hippocampus [27]. Interaction with the CB1 receptor has a modulating effect on GABAergic and Glutamatergic transmission [28], while also influencing the hypothalamic pituitary adrenal (HPA) axis, immune system activation, and neuroplastic mechanisms. In respect to specific psychotropic mechanisms of action, the anxiolytic (and antidepressant effects) may also in part be mediated via CBD’s serotonergic effects via 5-HT1A receptor activation [29], and THC’s CB1 receptor agonism [30, 31]. It is worth noting that studies have demonstrated that CBD may partially inhibit the psychoactive effects of THC, with CBD and THC having demonstrated differing symptomatic and behavioural effects on regional brain function [32–35].

As in the case of certain other psychiatric symptoms and disorders, epidemiological evidence indicates that there is a relationship between cannabis use and anxiety symptom levels. This association (assessed by Kelzior and colleagues [36] via meta-analysis of 31 studies) has to date only been found to be weak, and based largely on cross-sectional data. Thus, it may be that those with anxiety seek cannabis treatment, rather than a causal effect occurring from cannabis use. Longitudinal data is also not convincing due to the bias of one study with a large odds ratio included in their meta-analysis. However, a stronger positive association was revealed between anxiety and cannabis use disorder. Other longitudinal data involving the USA-based National Epidemiologic survey on Alcohol and Related Conditions [37] confirms there is no obvious causal inference. The study included individuals with a diagnosis of any anxiety disorder during the initial 4-year data collection period, comparing cannabis nonusers to users, and also individuals with cannabis use disorder at a later time point on a range of psychosocial measures. Results revealed that, when controlling for baseline confounders, no significant relationship was found with cannabis use and a greater frequency of anxiety.

While to date no human trials could be located for treatment of Generalised Anxiety Disorder using whole cannabis plant extracts or combined isolates, there was one study identified testing CBD (Table 1) for Social Anxiety Disorder. One small preliminary double-blind RCT compared the effects of a simulated public speaking test on treatment-naïve patients with social anxiety (*n* = 24) versus healthy control participants (*n* = 12) [38]. Each group received a single acute oral dose of CBD (600 mg) 1.5 h before the test, or matching placebo. Results revealed that pre-treatment with CBD significantly reduced anxiety, cognitive impairment and discomfort in the social anxiety group’s speech performance, and significantly decreased hyper-alertness in their anticipatory speech compared to the placebo group (which presented higher anxiety, cognitive impairment, discomfort, and higher alertness levels). Neuroimaging research has also revealed that in individuals diagnosed with social anxiety, cerebral blood flow may be altered via CBD. One study employed fMRI in 10 treatment-naïve patients with social anxiety who were given 400 mg of oral CBD or placebo in a double-blinded crossover manner. Relative to placebo, 400 mg of CBD was associated with significantly decreased subjective anxiety, with blood flow being modulated in the left parahippocampal gyrus, hippocampus, and inferior temporal gyrus, and the right posterior cingulate gyrus [39]. This suggests that CBD’s activity may occur via interaction with the limbic and paralimbic brain areas.

**Table 1:** Medicinal cannabis trials in mental disorders

Mental Disorder#	Cannabinoid(s) Studied	Methodology	Results	Clinical Comment
Social Anxiety				
Bergamaschi [38]	CBD (600 mg)	24 treatment-naïve patients with Social Anxiety were blindly allocated to receive CBD or placebo 1.5 h before a simulated public speaking test. 12 unmedicated healthy controls also completed the test. Self-reports on the Visual Analogue Mood Scale, and Negative Self-Statement scale, and physiological measures were taken at six time points during the test	Pre-test CBD administration in Social Anxiety patients versus placebo, resulted in significantly reduced anxiety, cognitive impairment and discomfort in speech performance, and significantly decreased hyper-alertness in anticipatory speech. CBD and control groups however did not differ, reflecting similar response profiles during the public speaking test	The initial positive studies suggest that CBD may be a beneficial safe option (a larger confirmatory study needed)
Crippa [39]	CBD (400 mg)	Compared regional cerebral blood flow activity in 10 treatment-naïve patients with SAD who were given CBD or placebo, in a double-blinded crossover manner	CBD compared to placebo, resulted in significantly lower subjective anxiety, and modulated blood flow in the left parahippocampal gyrus, hippocampus, and inferior temporal gyrus, and right posterior cingulate gyrus	
PTSD				
Greer [54]	Cannabis (not defined)	Analysed retrospectively collected CAPS data from 80 patients with PTSD	Patients reported > 75% decrease in CAPS scores when they were using cannabis compared to periods when they were not	No firm evidence yet, however initial case analyses suggest this application may be of benefit to manage PTSD symptoms, reduce anxiety, and improve sleep
Elms [53]	CBD (capsule or spray; mean dosage at week-8 of 49 mg)	Open label retrospective case study data from 11 adult patients with PTSD. Data assessed over 8 weeks	Mean PTSD symptoms on the PCL-5 reduced by 28%. Actual statistical data analysis not conducted	
Depression				
Portenoy [60]	Nabiximols: THC (2.7 mg) and CBD (2.5 mg)	263 patients with advanced cancer and opioid-refractory pain were randomly allocated to receive placebo or nabiximols daily at low (1–4 sprays), medium (6–10 sprays) or high (11–16 sprays) doses, for 5 weeks. Pre/post-measures included average pain, worst pain, sleep disruption, quality of life and mood	Reports of pain relief were significantly greater for nabiximols than placebo overall, especially in the low- and medium-dose groups. There were no other significant group differences. Adverse events were dose-related with only the high-dose group reporting a decrease in mood	No evidence for use in depression, however higher doses of THC-predominant medicines may in fact lower mood
Insomnia				
Shannon [69]	CBD capsules (25 mg) + liquid (6–25 mg)	Patient (10 y.o. girl with prior early childhood trauma) was prescribed fish oil (750 mg daily) + 1 CBD oil capsule daily for 5 months. CBD liquid (12–24 mg) was added to the regime for 1 month and reduced to 6–12 mg p.r.n (or ‘when needed’). Sleep assessed monthly via SDSC	SDSC scores decreased over the 5-month period, indicating an increase in sleep quality and quantity	Only case study and secondary outcome evidence at present. Encouraging as a potential use pending controlled studies, however next-day effects need to be assessed in terms of somnolence and cognitive functioning
Johnson [71]	Nabiximols: THC (2.7 mg) and CBD (2.5 mg) OR THC only (2.7 mg)	43 patients, with advanced cancer and opioid-refractory pain, self-administered daily nabiximols or THC-only sprays for 5 weeks. Safety, tolerability, pain and quality of life were assessed	Across groups, pain decreased at every visit, and showed pre−/post improvement with insomnia and fatigue	
Shannon [70]	CBD capsules (mainly 25 mg/day)	A retrospective case series of 72 adults given CBD for anxiety and sleep complaints at a psychiatric clinic, as an adjunct to usual treatment. Assessed monthly over 12 weeks	Anxiety scores on the HAMA decreased within the first month in 79% of the sample and remained decreased during the study duration. PSQI sleep score improved within the first month in 67%, but fluctuated over time. Data appeared to not be statistically significant for the group presenting with a primary complaint of anxiety (those with sleep disturbance fared better)	
Schizophrenia				
Leweke [99]	CBD (600–800 mg)	42 individuals with schizophrenia were randomly assigned to receive 600–800 mg of CBD or amisulpride over 4 weeks. The PANSS and BPRS were administered every 14 days. Blood was also collected	Both treatments were effective in reducing PANSS and BPRS scores at each time point. CBD was tolerated better, with fewer side effects reported. Anandamide levels were higher in the CBD group post-treatment	Avoid any use of high THC in youth. 600 mg–1200 mg of CBD per day may be effective as an adjunct for +ve and -ve symptoms
McGuire [101]	CBD (1000 mg)	88 antipsychotic-treated patients with schizophrenia were randomly given placebo or CBD alongside existing medication for 6 weeks. Pre/post-trial measures included the PANSS, Brief Assessment of Cognition in Schizophrenia, Global Assessment of Functioning, Clinical Global Impressions of Improvement and Severity scales.	The CBD group reported lower positive symptom scores, and were more likely to be rated as improved and less severely ill than the placebo group. The CBD group also showed improvements in the cognitive domain of motor speed compared to placebo. CBD was tolerated well with similar adverse event rates reported between the groups	
Boggs [102]	CBD (600 mg)	36 individuals with schizophrenia were randomised to receive CBD or placebo adjunctively to current antipsychotic medication for 6 weeks. PANSS and MCCB were assessed pre/post-trial	Both groups showed improvement on PANSS scores and only the placebo group improved on the MCCB. Similar side effects were noted between the groups, with more sedation evident in the CBD group	
Bipolar Disorder				
Zuardi [105]	CBD (600–1200 mg)	Two patients with bipolar I disorder were administered CBD for 30 days with 5 days of placebo pre/post-trial. Patients were assessed on the YMRS and BPRS every 7 days	One patient showed improvements in YMRS and BPRS scores while on CBD plus olanzapine but no additional improvement during CBD monotherapy. The second patient had no symptom improvement with any dose of CBD. Both tolerated CBD well with no side effects reported.	Not presently recommended. CBD appears not to be effective in attenuating mania
ADHD				
Cooper [108]	Nabiximols: THC (2.7 mg) and CBD (2.5 mg)	30 adults with ADHD were randomly prescribed nabiximols or placebo for 6 weeks. A participant’s optimal dose was decided at day 14. The QBT assessed cognitive performance and activity level (head movements), Conners Adult ADHD Rating Scale rated ADHD symptoms, and self-reports to examine emotional lability	The nabiximols group showed an improvement in QBT scores that approached significance. Nominally significant improvements in ADHD symptoms were also found for the nabiximols group compared to placebo	Potentially may be effective in managing some ADHD symptoms however more research is needed. Lower THC formulas alleviate concerns about cognitive impairment

# First Author; *THC* Tetrahydrocannabinol, *CBD* Cannabidiol, *QBT* Quantitative Behavioural Test, *PANSS* Positive and Negative Syndrome Scale, *MCCB* MATRICS Consensus Cognitive Battery, *HAMA* Hamilton Anxiety rating Scale, *PSQI* Pittsburgh Sleep Quality Index, *YMRS* Young Mania Rating Scale, *BPRS* Brief Psychiatric Rating Scale, *SDSC* Sleep Disturbance Scale for Children, *CAPS* Clinician Administered Posttraumatic Scale; PLC-5 = PTSD checklist for DSM-5

Due to the small sample sizes, the above data needs to be considered with caution. Further, appropriate and considered treatment of anxiety disorders with cannabinoid therapies is crucial due to the complex relationship with substance use disorders, often requiring a more complex biopsychosocial approach [40]. With this context in mind, CBD (being a non-intoxicant compared to THC) may be a more preferable option, having also shown anxiolytic effects in preclinical studies [41].

In respect to planned or ongoing research, one study in Colorado USA has just commenced and is exploring the anxiolytic effects of vaporised or ingested THC/CBD in differing ratios (1:0, 1:1, 0:1) in people with mild-moderate anxiety [42]. Another study is assessing the effect of CBD on reducing symptoms of anxiety disorders in a youth cohort (12–25 years old). The Australian-based study is a 12-week open-label pilot, which aims to see if 200 mg–800 mg of oral CBD (titrated depending on age, tolerability, and efficacy) is safe and effective for a youth population [43].

#### Post-traumatic stress disorder (PTSD)

Whole plant cannabis use for the management of PTSD symptoms has been identified in usage analyses [44], and in particular in returned armed services veterans [45]. The proposed neurobiological mechanisms by which medicinal cannabis may assist with PTSD are varied and mostly derived from animal research. There are high concentrations of endocannabinoid receptors in the prefrontal cortex, amygdala and hippocampus [46], having a role in fear acquisition and extinction [47]. There is strong evidence revealing that a disruption of the endocannabinoid system impairs fear extinction in CB1 knockout mice, suggestive of a critical role of CB1 receptors (and thereby potentially THC) being related to the extinction of fear [48–50].

One survey involving a convenience sample of 170 patients via a medical cannabis dispensary in California evaluated a range of health elements, the frequency of cannabis use, and general mental health [51]. Results revealed that those with high PTSD scores (assessed via The PTSD Checklist-Civilian Version) were more likely to use cannabis to assist with mental health coping, in addition to improving sleep, when compared with those with low PTSD scores. In particular, cannabis use frequency was greater among those with high PTSD scores who often used this for improving sleep. While there is increased use of cannabis in those with PTSD, there is currently no firmly supportive epidemiological data. A cross-sectional case control study of veterans showed that regular users do not have lower PTSD symptoms than non-users [52].

A recent open label retrospective analysis of case study data from 11 adults with PTSD assessed the patients over 8 weeks of CBD treatment (capsule or spray; mean dosage at week-8 of 49 mg) [53]. Results revealed that a reduction in mean PTSD symptoms occurred in 28% of the sample, as assessed on the PTSD checklist for DSM-5 (PCL-5). Statistical data analysis was not conducted, however, and thereby it is not possible to draw firm conclusions. Another retrospective study analysing PTSD symptoms collected during 80 psychiatric evaluations of patients applying to the New Mexico Medical Cannabis Program during 2009 to 2011 [54], revealed more supportive findings. The data identified a greater than 75% reduction in Clinician Administered Posttraumatic Scale for DSM-IV (CAPS) symptom scores when patients with PTSD were using cannabis compared to when they were not. While this study had a small sample, and is a retrospective analysis that has some methodological weaknesses, a 75% reduction on the CAPS is a compelling result, and has spurred recent RCTs which are currently in recruitment [55, 56].

#### Depression

Phytocannabinoids and terpenes have a potential application for modulation of the endocannabinoid system and the 5HT1A receptor to provide an antidepressant effect [16]. No RCTs to date have been conducted on the primary outcome of depression. Three studies assessing oral-administered nabiximols (i.e. botanically derived preparation containing standardised levels of THC, CBD, terpenes and flavonoids from cannabis) for other conditions (multiple sclerosis and cannabis withdrawal) found no significant effect on the secondary outcome of depression [57–59]. It is worth noting that one study involving cancer patients using nabiximols showed a significant *reduction* in mood occurred for those who used the highest dose (11–16 sprays per day) compared to the placebo [60]. Further, some epidemiological evidence has revealed a greater level of depressive symptoms in heavy cannabis users compared to light-users and non-users [61]. Due to this, higher dose THC should be avoided in people with major depressive disorder (MDD) or low mood. However, a cross-sectional survey on patterns of use and perceived efficacy suggested that in over 1429 participants identified as medical cannabis users, over 50% reported using medicinal cannabis specifically for depression [62].

### Insomnia

Anecdotal survey evidence abounds for the soporific effect of cannabis, with sufferers of a range of conditions including pain, anxiety and PTSD reporting that it assists in the management of insomnia [51, 63–68]. While this may commonly take the form of whole plant cannabis being administered via vaporised inhalation, isolated CBD may also be of benefit. An example case study detailed in the literature concerns a 10-year-old girl with prior early childhood trauma [69]. A trial of oral CBD oil (25 mg) resulted in a decrease in this patient’s anxiety, and improvement in the quality and quantity of her sleep. A more substantial retrospective case series of 72 adults given CBD for anxiety and sleep complaints at a psychiatric clinic (as an adjunct to usual treatment) assessed patient data monthly over 12 weeks [70]. Anxiety scores on the Hamilton Anxiety rating Scale (HAMA) decreased within the first month in 79% of the sample and remained low during the study duration. The Pittsburgh Sleep Quality Index score also improved within the first month in 67% of the sample, but fluctuated over time. It should be noted that the data were not analysed for statistical significance, and it appeared that the sub-sample presenting primarily for anxiety treatment did not fare as well as the cohort presenting primarily with sleep issues.

A study by Johnson et al. [71] tested the long-term safety and tolerability of a THC/CBD spray and a THC spray in relieving pain in patients with advanced cancer. A total of 43 patients were continued on a previous three-arm RCT involving an open label administration of a self-titrated THC/CBD spray (*n* = 39) or THC spray (*n* = 4) (2.7 mg) assessed over a 5-week period. While results revealed a consistent reduction in perceived pain, participants also reported a decrease in their insomnia, which also reflected less fatigue. Cannabinoids may have a dual effect of lessening pain (which makes it easier to sleep), in addition to their direct soporific and anxiolytic effects being mediated in part via serotonergic activity.

As detailed above, the evidence for this use is currently very weak, and to date no RCTs were located in the literature specifically assessing cannabinoid isolates or whole plant formulas. As of late 2018, there is however, a clinical trial taking place in Australia assessing cannabinoid treatment in chronic insomnia [72]. The study, based in Western Australia, is aiming to enrol 24 participants aged (25–70 years) who have insomnia (defined as difficulty initiating or maintaining sleep for 3 or more nights per week for at least 3 months). The intervention involves an oral MC extract (ZTL-101) or placebo given in a cross-over manner for a study period of approximately 2 months. Participants will be assessed via the clinically-validated insomnia scales, an actigraph watch, and will be assessed in a sleep centre after 2-weeks.

### Psychotic disorders

#### Schizophrenia

Consistent evidence has shown that there is a relationship between schizophrenia and cannabis use [73–75]. Heavy cannabis use may proceed to a diagnosis of the disorder, however, increased use may also result from ‘self-medication’. Cannabis use is cross-sectionally associated with more severe symptoms of psychosis in young people who do not meet the threshold for schizophrenia, and appears to be one high-risk component for the tumescence of the disorder [76]. More importantly, there is also longitudinal data to support a causal relationship [77–79]. A 2016 meta-analysis showed that while general lifetime use is not cross-sectionally associated with increased risk of psychosis, there is a robust relationship demonstrated in recent or current use in ultra-high-risk (UHR) adolescents with a DSM-diagnosed cannabis use disorder [80]. A recent prominent study has corroborated this finding. Data from 11 sites across Europe and Brazil involving patients with first-episode psychosis versus population controls, revealed that daily cannabis use was associated with increased odds of a psychotic disorder occurring compared with never-users, with nearly five-times increased odds for daily use of high-potency THC types of cannabis [81]. Several academics [82–86] have disputed these findings and comment that while there is a relationship, cannabis use is not causally related to increased psychosis risk (potentially due to a range of confounders e.g. correlated genetic liabilities or indirect and bidirectional processes). However, di Forti and colleagues (the study authors) [87] maintain that the data does indeed support this causal association, and that other research has flawed elements (e.g. previous Mendelian Randomisation studies using imprecise measurements of cannabis use).

It is of note that schizophrenia risk alleles are linked to cannabis use in a general population [78]. Regardless, the transition rates from a general population of cannabis users to schizophrenia is very low and can be considered to be part of a constellation of various potential gene-environment interactions. Several key genes have been implicated as potentially modulating the risk of developing schizophrenia after early cannabis use: BDNF, CNR1, COMT, AKT1, and DRD2 genes [88, 89]. There is also a likely increased susceptibility when a combination of these at-risk alleles from these single nucleotide polymorphisms (SNPs) are combined with childhood trauma [90, 91].

The apprehension regarding the promotion of psychotic symptoms are primarily based on the THC constituent of cannabis, a CB1 receptor agonist, which is the primary psychoactive phytochemical. This effect has been shown to be more prominent in users of high-THC cannabis, or in chronic heavy users [92]. Thus, THC should be avoided in people with or at risk of schizophrenia. Exposure to THC increases extracellular dopamine and glutamate and decreases GABA concentrations in the prefrontal cortex [93]. A recent double-blind crossover RCT investigated whether altered striatal glutamate (measured via proton magnetic resonance spectroscopy) was a mediating biomarker from intravenously administered THC in 16 healthy participants [94]. Results revealed that that an increase in striatal glutamate levels may underlie acute cannabis-induced psychosis, while lower baseline levels may provide a valid biomarker of greater sensitivity to its acute psychotomimetic effects.

The psychotropic effects of THC may mimic the presentation of psychotic symptoms, including paranoia, sensory alteration, euphoria, and hallucinations [95]. In laboratory-based research, people with schizophrenia appear to be even more sensitive to the psychosis-inducing effects of THC than healthy controls [96]. In contrast to THC, as mentioned in the introduction, CBD may in fact provide an opposing effect to THC albeit more research into this mechanism is required. Additional effects include the inhibition of anandamide breakdown via fatty acid amide hydrolase (FAAH) blocking effects, and anti-inflammatory effects [97, 98].

CBD is well-tolerated with minimal deleterious psychoactive effects (although some psychological effects are evident due to modulation of the 5HT1A receptor and enhanced anandamide signalling) [99]. Due to this, studies have primarily employed isolated CBD, however this work could potentially be extended to formulas from whole-plant strains which are high in CBD (> 10 mg/g) and lower in THC (< 4 mg/g). These preparations may also contain other yet-to-be-studied compounds from the plant which may be beneficial for the positive or negative symptoms of schizophrenia.

In respect to current research, aside from an initial index case study conducted by Zuardi, Morais [100] in 1995, who showed that 1500 mg of CBD administered for 26 days was beneficial for treatment-resistant schizophrenia, three clinical studies exist to date. A study by Leweke, Piomelli [99] tested in a double-blind, RCT design 600–800 mg/day of oral CBD vs the antipsychotic amisulpride over 4 weeks in 42 patients. While both treatments were safe and led to significant non-differential clinical improvements, the CBD arm had a superior side-effect profile. CBD also significantly increased anandamide levels, which was associated with clinical improvement. Another double-blind parallel-group trial, involving 88 patients with schizophrenia who were given either oral CBD (1000 mg/day) or placebo adjunctively to existing antipsychotic medication revealed after 6 weeks of treatment that the CBD group had lower levels of positive psychotic symptoms on the Positive and Negative Syndrome Scale (PANSS), and were more likely to have been rated as improved via clinician-ratings [101]. While these studies were supportive of CBD, a recent double-blind RCT by Boggs, Surti [102] found no benefit for 600 mg/day of CBD in comparison to placebo. The 6-week study involving 36 patients with schizophrenia revealed that both placebo and CBD PANSS scores improved, but no Group × Time effect was evident. The CBD was well-tolerated, however, and more sedation was evident in the CBD group compared to placebo.

Furthermore, CBD may confer some protective effects in young people at clinical high-risk for psychosis (*n* = 33), as a recent single-dose RCT found that 600 mg of CBD temporarily normalised aberrant brain activity in the parahippocampal, striatal, and midbrain areas, which is associated with increased psychosis risk [103]. Currently, an ongoing clinical trial in the United Kingdom is assessing the efficacy of 600 mg of CBD per day for reducing symptoms of psychosis in young people at clinical high-risk for psychosis [104].

#### Bipolar disorder

To date no clinical trial has assessed cannabinoids for the treatment of bipolar disorder (in respect to maintaining euthymia, or as a treatment of hypo/mania or depression), although there is a potential role of the endocannabinoid system in the disorder, as detailed above. Initial case reports contend this approach may not however be of benefit [105]. Two patients diagnosed with DSM-IV Bipolar type I disorder, and presenting with mania, were provided adjunctive CBD (titrated to 1200 mg per day) after receiving placebo for an initial five-day period. On Day 31, CBD treatment was discontinued and replaced by placebo for five days. While the first patient showed symptom improvement while on olanzapine plus CBD, she showed no additional improvement during CBD monotherapy, while the second patient had no symptom improvement with any dose of CBD during the trial. Both patients tolerated CBD very well and no side-effects were reported, despite no obvious effect on reducing mania.

### ADHD

Evidence has revealed that adults with ADHD may self-medicate with cannabis as a coping strategy for a range of potential effects [106]. Off-label use in the US for this application has been noted despite a relative deficit of evidence for this use [107]. One study was located, the “Experimental Medicine in ADHD-Cannabinoids” pilot RCT, using nabiximol (cannabinoid/terpene combination) oromucosal spray in 30 adults with ADHD for 6 weeks [108]. The primary outcome was cognitive performance and activity level (as measured by head movements) using the Quantitative Behavioural Test. Secondary outcomes included ADHD and emotional lability symptoms. While a trend towards significance occurred in favour of nabiximols, no significant difference was revealed on the primary outcomes. Notably, the use of nabiximols did not impair cognition. For secondary outcomes, the combination of note was associated with a nominally significant improvement in hyperactivity/impulsivity scores on the investigator-rated Conners Adult ADHD Rating Scale. The combination was well-tolerated, however, a serious adverse event involving muscular seizures/spasms occurred in the active group. While not definitive, this study provides preliminary evidence supporting the self-medication theory of cannabis use in ADHD and the need for further studies of the endocannabinoid system in ADHD. Results, however, did not meet significance following adjustment for multiple testing, and it should be recognised that the sample size was small, thus a more robust sample would be better placed to determine the true effect.

## Discussion

### Data synthesis

As the present data indicates, the current field of cannabinoid therapeutics in psychiatry currently provides no convincing evidentiary support for use in any mental health application. More research is urgently needed, and many RCTs are currently being undertaken; thereby the landscape will change rapidly over the next several years. Currently, the most promising (although inconclusive) evidence is for CBD as an adjunctive treatment in schizophrenia, with an additional isolated study showing efficacy in social anxiety, and weak data suggesting a potential effect for ADHD symptoms. The evidence also tentatively suggests that a role exists for cannabinoids in PTSD, and also in reducing insomnia, which may also commonly occur in chronic pain. For other plant-derived cannabinoid therapy applications for psychiatric symptoms/disorders (e.g. several affective disorders) no firm conclusions can currently be drawn.

### Clinical prescriptive considerations

It initially should be recognised by clinicians that, as detailed above, weak evidence currently exists in the field, thus this prescriptive advice should be taken in the context of evolving research. The first consideration faced by a clinician (in a legal jurisdiction) with a patient who is interested (or for clinician-initiated prescription) in using cannabis medicinally, is whether this is medically appropriate for them. A thorough screening firstly needs to occur, with Canadian British Columbian Physician guidelines [109] suggesting that clinicians initially assess:

- Age – higher-dose THC forms not advised in people < 25 years of age;
- If a personal history or family history of psychosis is present, and if so, no THC is advised;
- Any current or past drug or alcohol misuse or dependence (avoid especially in individuals with cannabis dependence or misuse);
- Cardiovascular or respiratory diseases (avoid or use caution);
- Current medications which may interact with cannabis; and
- Pregnant or planning or conceive or breastfeeding (avoid).

Next, if no contraindication is apparent, medical consideration can be given to what potential clinical application the MC may present for, and the cannabis formulation or isolate/s that may be appropriate for them. Given the complexity of MC whole-plant formulas (and the current challenge to standardise for batch-to-batch consistency), companies have primarily tested cannabinoid isolates and analogues. The most studied including nabiximols (Sativex), nabilone (Cesamet), and dronabinol (Marinol). While this may provide more pharmacological assurity, such an approach also negates the potential of unique genetic chemovars of cannabis which may provide specific therapeutic activity due to a complex synergistic interaction of constituents (known as the entourage effect). Patient preference may also be towards vaporisation of dried raw material [110]. To this end, specific prescriptive considerations need to be adopted, including:

- Determining patient preference regarding administration - vaporisation (via specific devices), inhalation via traditional smoking apparatuses, oral dose (i.e. capsule, oil or in some cases food product), sublingual via lozenges or sprays. Note that each has a different onset of action and half-life. Inhalants will provide a more instantaneous effect (due to the alacrity of THC decarboxylation), whereas oral forms will take longer e.g. 45–90 min to take effect. Both forms of administration may be advisable to provide flexible symptom management;
- Patient’s personality in terms of the effects of higher THC formulas. Avoidance of higher THC formulas should occur in youth and in those with paranoid personalities;
- Potential for abuse (with greater theoretical potential in vaporised/inhaled forms [which also carries additional general health consequences]);
- When the application should occur with respect to occupational and carer responsibilities and driving. Note that there is the potential to prescribe different cannabis preparations which contain differing levels of THC and CBD, with higher dose THC applications being applied preferentially in the evening.
- CBD dosage (based on current evidence) varies according to disorder, age, weight, and potentially pharmacogenetic differences. Most research tends to focus on a range of 200 mg–800 mg per day [111]. In respect to THC-containing formulas, it has been advised to be cautious exceeding 20 mg per day due to potential side effects [112], and people may find a psychotropic effect with as little as 1 mg–2.5 mg per dose.

As mentioned above, there are a myriad of potential cannabis chemovars that can be developed, each with unique medicinal applications. However, to maintain pharmacodynamic/kinetic consistency, at present, the two major constituents commonly standardised for are THC and CBD (in some cases select terpenes are also included). THC provides, as indicated above, the primary psychotropic effect, and higher doses may be preferable for the amelioration of pain and inducement of sleep [63]. Further, it may provide an acute mood elevating effect in some people, however as mentioned, this may also elicit symptoms of paranoia, anxiety, and cognitive impairment (and in higher doses may actually impair mood). This effect may potentially be opposed by CBD (and/or other less studied cannabinoids), however data is mixed as to this effect. Additionally, the findings are not clear cut, with users of cannabis (to treat anxiety) having a statistically significant preference for higher THC/lower CBD containing cannabis cultivars [113].

### Safety considerations

Clinicians needs to be aware that cannabinoid therapies may elicit a range of side effects. In respect to potentially expected side-effects from cannabinoid interventions, occasional adverse effects revealed in clinical trials include co-ordination problems, dizziness, disorientation, euphoria, drowsiness or fatigue, dry mouth, nausea and gastrointestinal upsets [10]. Due to this, regular monitoring is advised, especially when commencing treatment in cannabis-naïve patients.

The previously cited report conducted by the National Academy of Sciences [6] on the health effects of cannabis and cannabinoids cites limited evidence that cannabis use increases the rates of initiation of other psychoactive drugs. Additionally, while there are concerns over the relationship with schizophrenia, no firm evidence shows any association between cannabis use and the likelihood of developing bipolar disorder. Further evidence is suggestive that smoking cannabis on a regular basis is associated with cough and phlegm production, while limited evidence exists suggestive of a statistical association between cannabis use and ischaemic stroke and/or acute myocardial infarction. Evidence is noted to exist for the association between increased cannabis use frequency and progression to developing problematic cannabis use [6], as well as potential respiratory infections/disorders (especially in the use of poor-quality raw material). Clinicians need to balance these concerns together with the potential benefits, especially regarding the potential for lesser harm from other prescriptive or illicit options in patients managing psychiatric and pain conditions.

### Pharmacogenetic considerations

Increased attention to the influence of pharmacogenetics factors is advised, with several genes being identified that may differentially affect cannabinoid pharmacokinetics and pharmacodynamics. A recent review led by Hryhorowicz [114] characterised pertinent genes with relevant interaction with cannabis into three broad categories: Receptor genes (CNR1, CNR2, TRPV1, and GPR55), transporter genes (ABCB1, ABCG2, SLC6A) and pharmacokinetic/metabolism (CYP3A4, CYP2C19, CYP2C9, CYP2A6, CYP1A1, COMT, FAAH, COX2, ABHD6, ABHD12). Research into the pharmacogenomic influence is however nascent, with most of the focus being on the relationship with cannabis dependence (e.g. CNR1 receptor SNPs which shows no obvious association), or schizophrenia (COMT, DRD2 SNPs showing a stronger correlation) [81, 114]. Further exploration of FAAH SNPs differentially affecting people’s response to CBD is also of value (given its important role in inhibiting the degradation of anandamide).

### Occupational and public health considerations

Occupational health and safety issues also exist in consideration with medicinal cannabis users. Workplace safety concerns have been raised in relation to the potential for medicinal cannabis use to impair judgement and psychomotor skills, especially in relation to motor vehicle use, operation of fixed and mobile plants particularly heavy industrial machinery, and the potential for risk-taking behaviors and those working in safety sensitive positions [115, 116]. Employers have a ‘duty of care’ to provide safe and healthy workplaces, which includes the management of alcohol and drug use and their potential to create unsafe workplaces or practices. Workplace drug testing (WDT) is common in some industries including mining, transportation and correctional services [117]. Employees in building, transportation, maritime and mining operations cannot use drugs, legal or illegal, if they could impair their ability to safely undertake their duties [118]. However, the presence of a drug, or its metabolite, in a person’s system is not always proportional to cognitive impairment [119]. In addition, WDT does not discriminate between recreational or medicinal use and could place medicinal cannabis patients at risk of discrimination or unfair dismissal. Implementation of WDT should be balanced with a greater knowledge on the dose response relationship between cannabis-based medicines and their potential side effects.

Medicinal cannabis patients may also be subject to mobile drug testing in jurisdictions such as Australia. The salivary testing process is inefficient for assessment of cannabis related driving impairment because the tests can trace THC in saliva for days after consumption, long after any cognitive impairment has abated. The potential impact of medicinal cannabis on function will vary with dose, the length of usage (tolerance), route of administration (oral versus smoking), [120] and saliva THC levels are not direct measures of cognitive status. Further, the concentration of THC in urine does not correlate with cognitive function [120]. Conversely, in the US, a whole blood THC level of 5 ng/mL has been established as a legal limit for driving in states where cannabis has been legalised [121]. Guidelines and strategies for the specific risk management of cannabis in the workplace have been published in North America [120–123]. However, Australia is yet to publish its own risk management guidelines relating to medicinal cannabis in the workplace, although generic workplace alcohol and drug risk management guidelines could be adapted in the interim [118].

### Future research considerations

It should also be noted that the majority of studies assessing the effect of cannabis on cognitive function were undertaken with low potency THC strains (< 4% THC), [120] and further study is required for both high potency THC medicines, as well as THC-free medicines such as CBD [121]. Further, more research is needed on the dosage required (especially of CBD and lesser studied cannabinoids), the potential entourage effect, the pharmacokinetics, and the influence of pharmacogenetics on both metabolism of the cannabinoids and the pharmacodynamics. Novel trial designs are advised in some instances, particularly involving employing high-quality RCTs (or N-of-1 designs), to explore the potential benefits in psychiatric conditions.

While research is rapidly advancing, there is a challenge regarding the adequate blinding of medicinal cannabis studies (due to the obvious psychotropic effect, and lack thereof in cannabinoid-removed controls) [124]. This may be addressed via cannabis naïve participants with psychomimetic controls (e.g. atropine; these however have the innate challenges of being biologically active themselves); adequate assessment of un/blinding; and use of varying levels of THC within the study. It still should, however, be taken in the context of other psychiatric or neurological research, with opioids and benzodiazepines also eliciting an obvious psychophysiological effect, and the acceptance of the research demonstrating analgesic and anxiolytic effects, respectively.

## Conclusions

Currently the evidence is nascent and too weak to recommend cannabinoid-based interventions for a range of psychiatric disorders. While encouraging, research is only just beginning to determine whether cannabis or its isolates may or may not be effective for this application, and clinicians need to be mindful of several safety considerations (as articulated above). The most promising (although inconclusive) evidence is for CBD as an adjunctive treatment in schizophrenia, with an additional isolated study showing efficacy in social anxiety, and some data suggesting a potential effect for PTSD and ADHD symptoms. The data also tentatively suggests that a role exists for cannabinoids in reducing insomnia, which may also commonly occur in chronic pain. Given the generally favorable safety profile of cannabinoids observed across the observational studies and clinical trials conducted to date, there is clearly a strong case for encouraging further research.

## Data Availability

No data or materials were generated for this narrative review.

## Abbreviations

CBD: cannabidiol
HPA: hypothalamic pituitary adrenal
MC: medicinal cannabis
PANSS: Positive and Negative Syndrome Scale
PCL-5: PTSD checklist for DSM-5
SNP: single nucleotide polymorphism
THC: tetrahydrocannabinol
UHR: ultra-high-risk
WDT: workplace drug testing
